# Gram-Negative Bacterial Lysins

**DOI:** 10.3390/antibiotics9020074

**Published:** 2020-02-11

**Authors:** Chandrabali Ghose, Chad W. Euler

**Affiliations:** 1 Bioharmony Therapeutics, New York, NY 10016, USA; 2 Department of Medical Laboratory Sciences, Hunter College, CUNY, New York, NY 10065, USA; ceuler@hunter.cuny.edu; 3 Department of Microbiology and Immunology, Weill Cornell Medicine, New York, NY 10065, USA

**Keywords:** lysins, endolysins, bacteriophage, ESKAPE, Gram-negative, *Acinetobacter*, *Pseudomonas*, *E. coli*, *Klebsiella*

## Abstract

Antibiotics have had a profound impact on human society by enabling the eradication of otherwise deadly infections. Unfortunately, antibiotic use and overuse has led to the rapid spread of acquired antibiotic resistance, creating a major threat to public health. Novel therapeutic agents called bacteriophage endolysins (lysins) provide a solution to the worldwide epidemic of antibiotic resistance. Lysins are a class of enzymes produced by bacteriophages during the lytic cycle, which are capable of cleaving bonds in the bacterial cell wall, resulting in the death of the bacteria within seconds after contact. Through evolutionary selection of the phage progeny to be released and spread, these lysins target different critical components in the cell wall, making resistance to these molecules orders of magnitude less likely than conventional antibiotics. Such properties make lysins uniquely suitable for the treatment of multidrug resistant bacterial pathogens. Lysins, either naturally occurring or engineered, have the potential of being developed into fast-acting, narrow-spectrum, biofilm-disrupting antimicrobials that act synergistically with standard of care antibiotics. This review focuses on newly discovered classes of Gram-negative lysins with emphasis on prototypical enzymes that have been evaluated for efficacy against the major antibiotic resistant organisms causing nosocomial infections.

## 1. Introduction

The discovery of penicillin in 1928 at St. Mary’s Hospital in London changed the course of infectious disease treatment and increased patient survival [1]. Antibiotics have been the cornerstone of healthcare [2]. Unfortunately, antibiotic use and overuse has led to the rapid spread of acquired antibiotic resistance [3,4]. Antimicrobial resistance (AMR) is a major threat to global health and human development, and is not merely a medical problem, but also an economic and social problem [5]. Each year in the U.S. alone at least two million people are infected with antibiotic-resistant bacteria, and at least 35,000 of these patients die [6]. Multidrug-resistant (MDR) Gram-negative infections are widely recognized as one of the greatest areas of unmet medical need, particularly carbapenem-resistant pathogens such as *Klebsiella pneumoniae*, *Acinetobacter baumanni*i, and *Pseudomonas aeruginosa*, which are on the rise among hospitalized patients [5,7,8]. It is predicted that by 2050 more than 10 million people will die per year due to AMR infections unless action is taken today to spur development of novel and nontraditional antibiotics [9]. In this review, we discuss alternatives to antibiotics, namely bacteriophage lysins, for the treatment of Gram-negative bacterial infections.

## 2. Phages and Phage-Derived Molecules

Although bacteriophage therapy has been in use for at least the last century, the rising threat of AMR has renewed interest in phages and such phage-encoded products as antimicrobials and spurred the clinical development of such treatments in the last few years [10]. In the 1920s, d’Herelle, Eliava and their colleagues showed that phage therapy was effective for prophylaxis and treatment of skin infections caused by *Pseudomonas* and *Staphylococcus*, as well as diarrheal illnesses caused by *Salmonella* and *Escherichia coli* [11,12,13]. Although efficacy was shown in individual cases, the lack of consistent results and the timely discovery of small molecule antibiotics prevented a widespread adoption of phage therapy for clinical use in the western countries of the world [14]. Bacteriophage therapy is currently part of the antimicrobial armamentarium in Eastern European countries such as Georgia and Russia and is routinely used to treat different types of infections [15].

**Figure 1:**
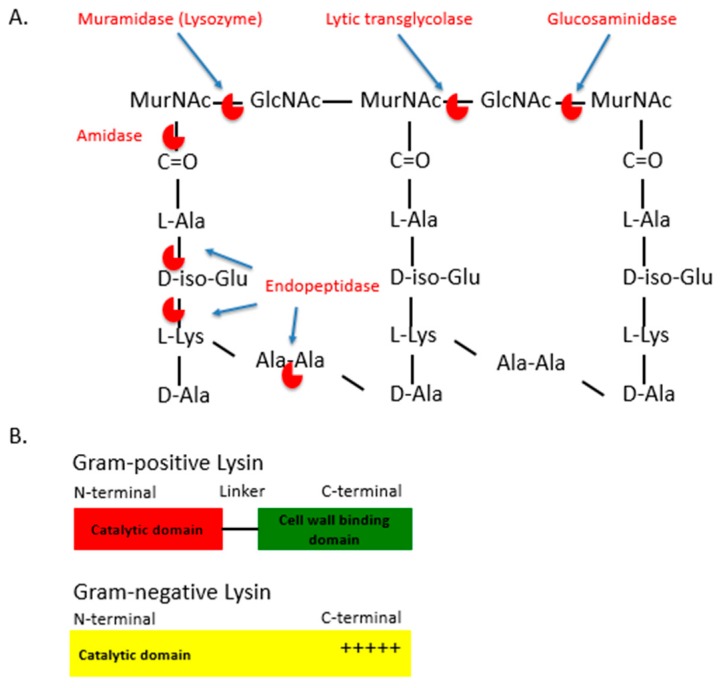
Structure and function of lysins. (**A**). Categories of lytic enzymes. Lytic enzymes are categorized based on the type of the chemical bond that is cleaved within the peptidoglycan layer. 1. Amidases hydrolyse the bonds between the sugar and the stem peptide moieties. 2. Endopeptidases cleave the bonds between amino acids of the stem peptides and the interpeptide bridges. Glycosidases, are subdivided into lysozymes or muramidases, lytic transglycosylases and glucosaminidases. Muramidases hydrolyse while lytic transglycolases cleave the same glycosidic bonds between the alternating MurNAc and GlcNAc disaccharide backbone. Glucosaminidases cleave glycosidic bonds between the GlcNAc and adjacent MurNAc monosaccharide in the glycan strand. (**B**). The schematic structure of a Gram-positive and a Gram-negative lysins.

In addition to phage therapy, phage products such as bacteriophage lytic enzymes are also under clinical development for the treatment of MDR infections [10,16,17]. Bacteriophage lytic enzymes, or lysins, are produced by bacteriophages during viral replication within bacterial hosts [18]. At the time of release of the progeny virions, lysins are produced along with other phage proteins such as holins [18]. These molecules lead to the disruption of the bacterial membrane and the cell wall, leading to bacterial rupture, death and the release of progeny virions. Lysins are capable of cleaving the major bonds in the peptidoglycan layer of bacterial cell walls. Lysins may be classified into five types, depending on the bonds these enzymes cleave in the peptidoglycan layer: (1) N-acetylmuramidases (e.g., lysozyme) and (2) lytic transglycolases cleave the N-acetylmuramoyl-β-1,4-N-acetyl glucosamine bond between the alternating MurNAc and GlcNAc disaccharide backbone of the peptidoglycan (Figure 1A); (3) N-acetyl-β-D-glucosaminidases cleaves the other glycosidic bond between the disaccharides (between the N-acetylglucosaminyl-β-1,4-N-acetylmuramine bond); (4) N-acetylmuramoyl L-alanine amidases, which cleave between the sugar and the stem peptide bonds, (e.g., N-acetylmuramoyl L-alanine bonds); (5) Endopeptidases, which cleave between amino acids (aa) of the stem peptides or the interpeptide bridges (Figure 1B) [19,20,21]. The peptidoglycan targets of these lysins are well-conserved and are often an essential component in the cell wall through the result of millions of years of evolution of the interactions between the bacteriophage and bacterial host, selecting for the efficient release of progeny virions from a phage-infected bacterium [19]. Lysins from bacteriophages that infect Gram-positive bacteria usually consist of one or two N-terminal catalytic domains (that carry out the different types of enzymatic cleavage described above) and a C-terminal cell wall binding domain (involved in the initial noncovalent binding to a specific target bacterium) (Figure 1B) [22,23,24]. While the cell wall binding domain is responsible for the high specificity of lysins, some Gram-positive catalytic domains are still very active when expressed on their own, with bacterium specificity then relying on the presence or absence of the targeted bonds in the cell wall. Likewise, Gram-negative lysins usually do not contain a cell wall binding domain, but are composed of an individual catalytic domain with terminal-charged residues for binding (Figure 1B) [22,25,26]. The independent activities and the modular nature of lysins allow for efficient catalytic or binding domain swapping with other lysins or antimicrobial components, fusion with antimicrobial peptides, etc., leading to the development of lysins that have enhanced bactericidal properties (Figure 1B) [22,25].

Given their targets and mechanism of action, resistance to lysins is orders of magnitude less likely than conventional antibiotics [27,28,29]. Such properties make lysins uniquely suitable for the treatment of MDR pathogens. Potential therapeutic lysins, when expressed recombinantly, are capable of “lysis from without” (LO) when applied to target bacteria externally. LO describes the biological phenomenon where either virion adsorption or the action of exogenous (applied from outside the cell) phage lysins causes bacterial cell lysis and death of the bacteria [30].

Lysins have fast-acting bactericidal activity and have the ability to kill bacteria immediately upon contact [31]. Minimal resistance to lysins has been observed to date in in vitro studies [31]. Another key feature that distinguishes lysins from many standard-of-care (SOC) antibiotics is their narrow-spectrum of activity that allows the targeting of pathogenic antibiotic-resistant bacteria, as well as those that are antibiotic-sensitive, while sparing the healthy host microbiome and preventing dysbiosis [32]. The potent, narrow-spectrum, rapid bactericidal and biofilm-disrupting effects of lysins make them promising antimicrobials [22,31,33,34].

In the last twenty years, numerous lysins have been identified and recombinantly expressed to target a number of Gram-positive bacteria such as *Bacillus anthracis*, methicillin-resistant *Staphylococcus aureus* (MRSA), *Clostridium difficile*, etc. [22,32,34,35,36]. Exebacase, an anti-staphylococcal recombinant lysin is the first lysin in the United States to currently undergo human clinical trials to treat *S. aureus* bacteremia and endocarditis [37]. A Phase 2 superiority study evaluating the safety, efficacy and tolerability of Exebacase in patients with MRSA bacteremia has shown a 42.8% improvement in the patient group receiving Exebacase plus SOC antibiotics compared to SOC alone [38]. A Phase 3 clinical trial of Exebacase for the treatment of patients with MRSA bacteremia, and/or right-sided *S. aureus* endocarditis, is slated to begin at the end of 2019.

## 3. Lysins for Gram-Negative Bacteria

Due to the structural barrier and the low permeability of the bacterial outer cell membrane, recombinantly expressed lysins have historically failed to gain access and cleave the underlying peptidoglycan to efficiently kill Gram-negative bacteria. The Gram-negative bacterial cell envelope is composed of protein, sugars and complex lipid arrangements [39]. Different approaches have been employed to increase the membrane permeability to lysins, such as the simultaneous addition of membrane destabilizing agents like poly-l-lysine, polymyxin B, or ethylene diamine tetraacetic acid disodium salt (EDTA), or modification of the lysins themselves, to include highly charged/hydrophobic aa residues [25,40,41,42,43] (Table 1). The success of these approaches has been varied, with few showing in vivo efficacy in animal models (Table 1) [22].

### 3.1. A. Baumannii Lysins

Carbapenem-resistant *Acinetobacter* causes at least 8500 infections per year in the USA, with an estimated 700 deaths in 2017 alone, and is considered to be an urgent threat by the Centers for Disease Control and Prevention [6]. *A. baumannii* has also received a Priority-1 classification by the World Health Organization for the development of new antibiotics, as few treatment options exist for infections caused by this MDR pathogen. Therefore, the development of new antimicrobials against *A. baumannii* are much needed, especially ones with novel mechanisms of action, like lysins. Multiple *A. baumannii*-specific lysins have been discovered, with a few showing the potential to be developed into a therapeutic agent.

LysAB2, an *A. baumannii*-specific lysin, was isolated from *A. baumannii* lytic phage ΦAB2 and has sequence homology to both glycoside hydrolases and to the muramidase/lysozyme-like superfamily [44]. Unlike Gram-positive lysins, Gram-negative lysins such as LysaAB2, are usually composed of an N-terminal lysozyme-like domain and a C-terminal positively charged region (Figure 1B). The C-terminal region of LysAB2 is composed of basic aa that are thought to bind to negatively-charged lipopolysaccharides (LPS) present on the bacterial outer membrane (OM), following which the N-terminal catalytic domain cleaves the bonds between the alternating N-acetylglucosamine and N-acetylmuramic acid of the bacterial peptidoglycan, leading to the lysis of the bacterium. LysAB2 has broad in vitro bactericidal activity towards both Gram-negative (MDR *A. baumannii* and *E. coli*) and Gram-positive (*S. aureus*) bacteria [44]. While the efficacy of LysAB2 in in vivo animal models of *A. baumannii* infection still remains to be elucidated, the bactericidal properties of antimicrobial peptides (AMPs) extracted from the C-terminal of lysin LysAB2 have been tested in vitro and in vivo against MDR *A*. *baumannii* strains [44]. LysAB2 P3 was the peptide derivative that showed the highest level of antibacterial activity against *A*. *baumannii*, possibly due to alterations in the aa sequence that increased the net positive charge and decreased hydrophobicity of the peptide [45]. In an in vivo intraperitoneal (ip) infection model, LysAB2 P3 reduced *A*. *baumannii* CFU by 13-fold in ascites and 27-fold in blood and was able to rescue 60% of the bacteremic mice [45].

PlyF307, a naturally occurring 16kDa lysin, was identified from an environmental strain of *A. baumannii* using a broad expression-based screening approach [22]. In contrast to most two-domain Gram-positive lysins (Figure 1B), PlyF307 is composed of an individual muramidase enzymatic domain with a C-terminal, aa 108-138, that has a high positive net charge. Biochemical characterizations of PlyF307 and *A. baumannii* interactions have indicated that the target site of PlyF307 is located on both the OM and inner membrane (IM) of *A. baumannii*. In Gram-negative bacteria the outer leaflet of the OM is composed of polyanionic lipopolysaccharides (LPS) that are stabilized by divalent cations (Ca2+ and Mg2+). At higher pH and lower salt concentrations, the positively charged aa of PlyF307 are thought to establish ionic interactions with the OM and initiate the lytic process by providing the N-terminal enzymatic domain access to the underlying peptidoglycan, leading to the disruption of the IM and eventually, bacterial cell death [22].

PlyF307 has significant in vitro bactericidal activity against clinically relevant *A. baumannii* strains [22]. A 2 h incubation of 10^6^ cfu/mL of *A. baumannii* with 100 μg/mL of PlyF307 resulted in a 5 log_10_ CFU/mL decrease in >80 clinical and environmental *A. baumannii* isolates. Additionally, while PlyF307 had no significant activity against *Pseudomonas* or *Staphylococcus*, a >2 log_10_ CFU/mL decrease was also observed against *E. coli* strains under the same conditions [22].

Due to the complex composition of bacterial biofilms, conventional antibiotics are generally unable to penetrate or degrade biofilms, or kill dormant bacteria harbored within the biofilms. As such, infections caused by biofilm-embedded bacteria like *A. baumannii* have become a major concern as they are more recalcitrant to treatment [61]. Luckily, a number of lysins have been shown to effectively destroy biofilms and kill the associated bacteria within these matrices [62]. The potency of PlyF307 has been demonstrated against *A. baumannii* biofilms formed on polyvinyl chloride (PVC) catheters [22]. A single, 2 h treatment with 250 μg of PlyF307 resulted in a >1.7 log_10_ CFU/mL decrease of the biofilm on the catheters. Additionally, observation of the catheters by scanning electron microscopy showed PlyF307 also destroyed much of the extracellular polymeric matrix of the biofilm. Furthermore, to simulate in situ treatment of indwelling catheters in patients, catheters with 2-day old biofilms were surgically implanted beneath the skin of mice. After 24 h, two doses of PlyF307 were injected subcutaneously to the site of the implant within a 4 h period. A 2 log_10_ CFU/mL reduction in *A. baumannii* viability under such experimental conditions suggests that it may be possible to treat infected implants with lysins as an alternative to surgically removing them.

PlyF307 is the first Gram-negative lysin to show in vivo efficacy as a therapeutic agent in a murine *A. baumannii* bacteremia model [22]. *A. baumannii* (10^8^ cfu) were ip injected to allow the mice to develop systemic bloodstream infection within 2 h, at which time a single therapeutic dose of PlyF307 (1 mg/mouse) was delivered by ip injection. PlyF307 treatment saved 50% of the bacteremic mice, while nearly all of the control mice died within 24 h.

A similar phage–genome-based screening approach was used to identify PlyE146, encoded by an *E. coli* prophage. The recombinant lysin was shown to have N-acetylmuramidase activity against several Gram-negative bacteria, such as *E. coli*, *P. aeruginosa* and *A. baumannii* [46]. PlyE146 exhibited the greatest level of bactericidal activity against *A. baumannii*, resulting in >5 log_10_ CFU/mL decrease in 2 h compared to >3 log_10_ CFU/mL decrease against the *E. coli* strains tested. Similar to the abovementioned lysins, PlyE146 contains a highly cationic C-terminal domain that may play a central role in the observed potent bactericidal activity [22]. This activity may have also been aided by the presence of a 6-His aa tag, which increased the positively charge residues on the C-terminal. PlyE146 has a slower rate of killing than PlyF307, whereby the killing effects are observed after 30 min of exposure [22]. Additionally, PlyE146 activity is inhibited in the presence of serum, thus unlike PlyF307, it may have limited feasibility as a potential systemic treatment for *A. baumannii* [22]. The efficacy of PlyE146 still remains to be tested in in vivo animal models of *A. baumannii* infection.

Additional *A. baumannii* specific lysins have been identified and recombinantly expressed, such as LysABP-01, PlyAB1, ABgp46, and Ply6A3, which may be promising and may potentially be developed for the treatment of MDR infections of *A. baumannii* (Table 1) [22,47,48,50]. Lysin LysABP-01 isolated from *A. baumannii* lytic bacteriophage ABP-01 has bactericidal activity against *A. baumannii*, *E. coli* and *P. aeruginosa* [47]. LysABP-01 has synergy with various antibiotics such as colistin, an antibiotic of last resort that is used to treat MDR infections [47]. Another lysin, PlyAB1, was isolated from *A. baumannii* bacteriophage ABp1, has bactericidal activity against 48 hospital-derived pan drug-resistant *A. baumannii* isolates [32,48]. Lysin ABgp46 isolated from *A. baumannii* phage Øvb_AbaP_CEB1 has bactericidal activity against several multidrug-resistant *A. baumannii* strains [49]. In the presence of membrane permeabilizing agents such as citric acid and malic acid, ABgp46 was active against additional Gram-negative pathogens such as *P. aeruginosa* and *Salmonella enterica* serovar Typhimurium. Lysin Ply6A3 produced by bacteriophage PD-6A3 was shown to kill 70% of MDR *A. baumannii* strains and protect 70% of mice in a lethal *A. baumannii* sepsis challenge model [50].

### 3.2. P. Aeruginosa Lysins

MDR *P. aeruginosa* nosocomial infections led to 32,600 cases and 2700 deaths in 2017 [6]. Such infections manifest as urinary tract and surgical site infections to more complicated pneumonia and bloodstream infections. Numerous strains of *P. aeruginosa* are resistant to many different classes of antibiotics, including the broadly used carbapenems [63]. Lysins that effectively kill *P. aeruginosa* could be useful in controlling the spread of *P. aeruginosa.*

Using an in silico approach to search *P. aeruginosa* phage genomes, the Fischetti laboratory has identified 16 *P. aeruginosa* specific lysins that had phylogenic similarities to *A. baumannii* lysin PlyF307 [22,51]. Of the 16 identified lysins, two lysins have shown promise as drug candidates: PlyPa03 and PlyPa91. Both lysins have displayed bactericidal properties against a number of clinical strains of *P. aeruginosa*, including biofilm-embedded strains. Unfortunately, both lysins were serum sensitive, thus limiting their use as a systemic therapeutic. However, PlyPa91 showed in vivo efficacy in a mouse model of *P. aeruginosa* skin infection, reducing bacterial CFUs by >2 log_10_s. PlyPa03 also had efficacy in a mouse model of *P. aeruginosa* pneumonia, where 70% of mice treated with two intratracheal and intranasal doses of PlyPa03 survived [34].

Additional lysins with antipseudomonal activity have been identified and tested mostly in in vitro bactericidal assays in the presence of OM permeabilizers (Table 1) [25,41,42]. Lysin gp144 (KZ144), isolated from the *P. aeruginosa* phage phiKZ, has one of the few Gram-negative modular lysins. It has an N-terminal peptidoglycan binding domain and a C-terminal catalytic domain with transglycosylase activity [42]. Gp144 was able to degrade chloroform-treated *P. aeruginosa* and, to a lesser extent, *S. aureus* and *B. cereus.* Lysin EL188, isolated from *P. aeruginosa* phage EL, has potent bactericidal activity in combination with OM permeabilizers such as EDTA [41]. A combination of EL188 and EDTA led to a >4 log_10_s reduction of *P. aeruginosa* in 30 min. While the potential use of EL188 was diminished by the need of co-administration of EDTA or similar chemicals, this combination may be suitable for use on local topical infections, such as those in burn patients [41]. LysPA26 was isolated from phage JD010. Based on sequence analysis, LysPA26 was shown to be part of a lysozyme-like domain family of superfamily cd00442 [25]. LysPA26 was capable of killing biofilm-embedded *P. aeruginosa* without the addition of outer-membrane permeabilizers and also has bactericidal activity against to *K. pneumoniae*, *A. baumannii* and *E. coli* [25]. To date, in vivo studies have not yet been performed to look at the therapeutic potential of these lysins.

### 3.3. Lysins Against Other Gram-Negative Pathogens

Additional recombinant bacteriophage lysins that have targeted other Gram-negative pathogens have shown mostly in vitro bactericidal activity, such as those against *K. pneumonia*, *E. coli*, *Burkholderia cenocepacia* and *S*. *enterica* ser. Typhimurium (Table 1). Lysin KP27, isolated from environmental phage vB_KpnM_KP27, has bactericidal activity against multidrug-resistant *K. pneumonia* [26,52]. AP3gp15, a muralytic enzyme originally encoded on the *Burkholderia* AP3 phage, has broad in vitro antibacterial activity against *B*. *cenocepacia*, as well as *E*. *coli*, *K*. *pneumoniae*, *P*. *aeruginosa*, and *S*. *enterica* ser. Typhimurium [26,52].

Several *E. coli* lysins have been identified and recombinantly expressed to date, such as the T5 lysin (EndoT5) and Lysep3 [43,53]. EndoT5, a peptidoglycan hydrolase belonging to the M15 family of peptidases, was able to lyse *E. coli* strains in vitro in the presence of various outer-membrane permeabilizers such as polymyxin B, gramicidin D, poly-l-lysine, chlorhexidine and miramistin [43]. Lysep3, a coliphage lysin isolated from the *E. coli*-specific bacteriophage vB_EcoM-ep3, has in vitro bactericidal activity against *E. coli* and *P*. *aeruginosa* [53]. Many modifications have been made to Lysep3 to increase its ability to penetrate the outer membrane. Lysep3 bactericidal activity was enhanced by fusing the *Bacillus amyloliquefaciens* bacteriophage lysin binding domain D8 to the C-terminal region of Lysep3 [54]. Additional variations include Lysep3 fusions with Colicin A, addition of positively charged aa to increase the number of positive charges and hydrophobic residues on the N or C-terminal domains, etc. [54]. The following section describes further methods used to modify lysins so as to enhance the therapeutic potential of these molecules to effectively kill Gram-negative bacteria.

## 4. Engineered Phage Lysins and Derivatives

While some naturally occurring Gram-negative lysins have potent in vitro and in vivo activity against the target bacterium, a number of strategies have been used to increase the intrinsic bactericidal activity by modifying the lysin’s ability to permeate the outer membrane, and to overcome serum inactivation.

### 4.1. Artilysins

Artilysins are fusion proteins that contain lysins fused to an OM destabilizing peptide that are active against both Gram-negative and Gram-positive pathogens [55]. One example of artilysins is Art-175, a broad-spectrum artilysin composed of a modified version of the *P. aeruginosa* lysin KZ144, which was fused to a potent AMP, sheep myeloid 29 aa peptide (SMAP-29) at its N-terminal [56,57,58]. The SMAP-29 peptide leads to the destabilization of the OM, allowing the enzymatic domain of KZ144 to gain access to and degrade the underlying peptidoglycan. Additionally, Art-175 was bactericidal against *A. baumannii*, *P. aeruginosa* and *E. coli* and leads to a reduction of >4–5 log_10_ CFU/mL in 30 min in an in vitro bactericidal assay [57]. Art-175 has shown efficacy in an in vivo mouse sepsis model of *P. aeruginosa* by rescuing 80% of challenged mice compared to the untreated controls [64].

### 4.2. Lysocins

Lysins may be engineered to penetrate and kill Gram-negative bacteria by exploiting colicin-like bacteriocins, which actively transverse the bacterial OM to deliver lysins to the peptidoglycan in the periplasmic space [34,65,66]. The first successful example of this type of hybrid lysin was the fusion of a binding domain from pesticin, a FyuA outer membrane transporter binding protein from *Yersinia pestis*, to the N-terminus of T4 bacteriophage lysozyme. This Pesticin–T4 lysozyme toxin was able to be transported across the outer membrane and kill *Y. pestis* in vitro, as well as other *Yersiniae* and *E. coli* isolates expressing the FyuA receptor [65]. Similarly, the Lysep3 coliphage lysin described above was also further modified to include the receptor binding and translocation domains of Colicin A, allowing it to translocate into the periplasmim and kill *E. coli isolates* from farms in in vitro assays [66] Furthermore, this hybrid lysin also reduced the amount of fluorescent *E. coli* in a mouse intestinal infection model. Recently, to address the concerns of MDR *P. aeruginosa*, another proof of concept study was initiated to fuse the *P. aeruginosa* phage PAJU2 muramidase GN4 to the *P. aeruginosa* bacteriocin pyocin S2 (PyS2) [34].

PyS2 consists of a N-terminal receptor-binding domain, a C-terminal DNAse domain, and a α-helical domain linked to an *E. coli* colicin-like domain [67,68]. PyS2 binds to ferripyoverdine receptor FpvAI, an iron transporter that was upregulated in an iron-depleted environment to allow for the transport of siderophore ferripyoverdine [69,70]. Following the PyS2–FpvA1 interaction, PyS2 passes through the iron channel to bind to TonB1, which is part of the iron transport Ton system, and allows PyS2 to translocate the OM. By replacing the DNAse domain of PyS2 with GN4 lysin the fusion allows the GN4 domain to translocate across the membrane and degrade the peptidoglycan of *P. aeruginosa* [34].

While effective in the presence of human serum, the 76 kDa hybrid lysin has a narrow spectrum of activity against *P. aeruginosa* strains expressing FpvAI type receptors, which account for a third of all clinical strains, thus restricting the broad application of this particular lysocin fusion protein [34]. Antipseudomonal activity was also tested in an in vivo murine model of lethal *P. aeruginosa* bacteremia and was shown to have dose-dependent efficacy, protecting from 73% to 100% of mice compared to 40% of untreated mice. Additional studies to derive lysocins that have broad-spectrum activity against all *P. aeruginosa* strains would be useful, as well as the development of lysocin derivatives that can target additional MDR Gram-negative pathogens.

### 4.3. Additional Modified Phage Lysins to Overcome Serum Resistance

A majority of Gram-negative peptide lysins mentioned above do not have bactericidal activity in physiological conditions containing serum. Therefore, lysins have been engineered to overcome serum resistance. A screen of 500 prophages and phage lysins was utilized to identify naturally occurring lysins that have bactericidal activity against *P. aeruginosa* and are active in the presence of human serum under physiological conditions [71]. Additionally, lysins with intrinsic antipseudomonal activity were further modified then screened for increased activity in serum using an agar-overlay assay supplemented with human serum. Synergy with SOC antibiotics, antibiofilm activity in the presence of human bodily fluids, and safety (hemolytic activity against human cells) were assayed to identify lysins that have the therapeutic potential for further development. CF 370 was identified as a lead lysin candidate for the treatment of *P. aeruginosa* [59]. In a rabbit pneumonia model, a single dose of CF-370 alone or in combination with meropenem led to a significant reduction of the bacterial load in the lungs, kidneys and spleen, as well as longer survival compared to untreated controls. Furthermore, CF-370 was well-tolerated and showed no adverse clinical effects in the treated animals [59].

## 5. Peptide Lysins

Engineered and naturally occurring antimicrobial peptides that are derived from bacteriophage lysins are described in the following section. While also being potent antimicrobials, most of these peptides are inactive in the presence of serum and serum components and therefore, in their current state, may only be able to be used topically to treat infections [31].

### 5.1. P307SQ-8C

Engineered lysin derivatives of *A. baumannii* lysin PlyF307, such as P307SQ-8C, are under development for the treatment of MDR *A. baumannii* infections via local delivery directly to the site of infection, such as the skin, lungs and bladder [22,31]. P307SQ-8C, a 39 aa non-toxic peptide that contains aa 108–138 of PlyF307, fused to a highly cationic 8 aa domain of a Hepatitis B virus core protein, which has intrinsic antibacterial activity [72]. While PlyF307, the parent full-length lysin, has activity in the presence of serum, the engineered lysin peptide derivative P307SQ-8C is inactive in the presence of serum and serum components [22,31]. P307SQ-8C has potent bactericidal activity against MDR and susceptible strains of *A. baumannii*, it eradicates bacteria in biofilms, and acts synergistically with antibiotics [31]. Furthermore, P307SQ-8C has bactericidal activity against over 100 clinical *A. baumannii* strains, including carbapenem-resistant strains and many that are polymyxin-resistant (unpublished data). When tested under alkaline conditions, at pH ≥ 8.0, the bactericidal specificity was broadened to also include *K. pneumoniae* and *E. coli* [31,72]. P307SQ-8C has comparable bactericidal activity, as measured by minimum inhibitory concentrations (MIC), to levofloxacin and ceftazidime and acts synergistically with polymyxin B against *A. baumannii*. Like PlyF307, P307SQ-8C was also able to disrupt *A. baumannii* biofilms embedded in PVC catheters [22]. In an in vivo murine skin infection model, a single treatment of P307SQ-8C was able to decrease the *A. baumannii* bioburden by >2 log_10_ CFU/mL [72]. P307SQ-8C represents a viable therapeutic option to the current systemically delivered antibiotics, which are either used sparingly due to the risk of developing resistance or are completely ineffective at reaching the local sites of infections.

### 5.2. Amurins

Amurins are a group of phage-encoded lytic antimicrobial peptides with different activities, which have potent broad-spectrum bactericidal activity against Gram-negative bacteria, and the ability to clear biofilm-embedded bacteria [73]. They also must have synergistic activity with many standard-of-care antibiotics (SOC) [60]. Given these attributes, amurins may be used to treat patients who are infected with biofilm-associated bacteria, such as those seen in cystic fibrosis patients, burn victims, and patients with in-dwelling catheters or who are on ventilators. In an in vitro proof of concept study, the activity of amurin peptide App2-M1 was tested against *Stenotrophomonas maltophilia* biofilm-containing hemodialysis catheters. *S. maltophilia* is an emerging opportunistic pathogen that is associated with nosocomial infections with a high mortality rate [59]. App2-M1 was successful in removing the biofilm at a concentration of 1 µg/mL, whereas meropenem, a commonly used carbapenem antibiotic, failed to eradicate the biofilm-embedded bacteria. This suggests the feasibility of developing amurins as a potential therapeutic for eradicating biofilm-associated Gram-negative bacteria. In vivo efficacy of Amurins has yet to be established.

## 6. Conclusions

As AMR continues to grow as a global health threat, novel and nontraditional therapeutic agents are urgently needed to fill the gap that traditional small molecule antibiotics are failing to meet. Common lysin characteristics such as a narrow spectrum activity, coupled with potent and rapid bactericidal activity against specific bacterial pathogens, anti-biofilm activity, a low propensity for developing resistance, and synergy with standard-of-care antibiotics, make these Gram-negative lysins ideal candidates to take on the challenge of AMR. As some of the more advanced Gram-negative lysins move into preclinical development, concerns will have to be addressed regarding immunogenicity, toxicity due to the release of LPS during the bactericidal killing process, and pharmacokinetic issues due to the complexity of the Gram-negative cell wall. The Gram-negative lysins summarized in this review are still in the early stages of development, but the initial in vitro and in vivo data support the hypothesis that Gram-negative lysins are potential antimicrobial agents that deserve to be explored further [10]. With more than 10^31^ phages on Earth, there is a staggering potential to identify new lysins to kill most, if not all, Gram-negative pathogens [30]. Furthermore, sophisticated assays and engineering methods are currently being developed for the easier identification and improvement of synthetic lysins and/or peptide derivatives that may display clinically superior antimicrobial potency compared to the naturally occurring lysins. Thanks to millions of years of bacteriophage–bacterial host evolution, bacteriophage lysins and their derivatives are ideally positioned to address the global AMR issue.

## Figures and Tables

**Table 1:** Characteristics of Gram-negative Lysins.

Lysin	Predicted Enzymatic Activity	Antimicrobial Spectrum *	In Vivo Efficacy	Reference
LysAB2 and derivatives LysAB2 P3	Lysozyme (Muramidase)	*A. baumannii*, *S. aureus*, *E. coli*	Bacteremia	[44,45]
PlyF307 and derivatives P307SQ-8C	Lysozyme (Muramidase)	*A. baumannii*, *E. coli*, *K. pneumonia*	Bacteremia, skin infection	[22,31]
PlyE146	Lysozyme (Muramidase)	*E. coli*, *A. baumannii*, *P. aeruginosa*,		[46]
LysABP-01	Lysozyme (Muramidase)	*A. baumannii*, *P. aeruginosa*, *E. coli*		[47]
PlyAB1	Glycosidase	*A. baumannii*		[32,48]
ABgp46	Glucosaminidase	*A. baumannii*, *P. aeruginosa*, *S. enteric*aser. Typhimurium		[49]
Ply6A3	Lysozyme (Muramidase)	*A. baumannii*, *E. coli*, *P. aeruginosa*, *K. pneumoniae*, *MRSA*	Sepsis	[50]
PlyPa103 and PlyPa91	Lysozyme (Muramidase)	*P. aeruginosa*	Skin infection, pneumonia	[51]
gp144 (KZ144)	Transglycolase	*P. aeruginosa*, *S. aureus*, *B. cereus*		[42]
EL188	Transglycolase	*P. aeruginosa*		[41]
LysPA26	Lysozyme (Muramidase)	*P. aeruginosa*, *A. baumannii*, *E. coli*, *K. pneumonia*		[25]
KP27	Lysozyme (Muramidase)	*K* *. pneumonia*		[26]
AP3gp15	Lysozyme (Muramidase)	*B. cepacia*, *E. coli*, *K. pneumonia*, *S. enteric*a ser. Typhimurium		[52]
EndoT5	Lysozyme (Muramidase)	*E. coli*		[43]
Lysep3 and derivatives	Lysozyme (Muramidase)	*E. coli*, *P. aeruginosa*, *Streptococcus sp.*	Gastrointestinal infection	[53,54]
Art-175 and Art-085	Transglycolase	*P. aeruginosa*, *A. baumannii*, *E. coli*	Skin infection, sepsis	[55,56,57,58]
Lysocins	Lysozyme (Muramidase)	*P. aeruginosa*	Bacteremia	[34]
GN 121 and CF370	Unknown	*P. aeruginosa*	Pneumonia	[59]
Amurin APP2-M1	Unknown	*S. maltophilia*		[60]

* Organisms in bold depict the primary organism that the bactericidal activity of the lysin was tested against.
